# Ethics of conducting research in conflict settings

**DOI:** 10.1186/1752-1505-3-7

**Published:** 2009-07-10

**Authors:** Nathan Ford, Edward J Mills, Rony Zachariah, Ross Upshur

**Affiliations:** 1 Médecins Sans Frontières, Johannesburg, South Africa; 2 Simon Fraser University, Vancouver, Canada; 3 British Columbia Centre for Excellence in HIV/AIDS, University of British Columbia, Canada; 4 Médecins Sans Frontières, Brussels, Belgium; 5 Joint Centre for Bioethics, University of Toronto, Canada

## Abstract

Humanitarian agencies are increasingly engaged in research in conflict and post-conflict settings. This is justified by the need to improve the quality of assistance provided in these settings and to collect evidence of the highest standard to inform advocacy and policy change. The instability of conflict-affected areas, and the heightened vulnerability of populations caught in conflict, calls for careful consideration of the research methods employed, the levels of evidence sought, and ethical requirements. Special attention needs to be placed on the feasibility and necessity of doing research in conflict-settings, and the harm-benefit ratio for potential research participants.

## Introduction

Despite the fact that conflicts are widespread in several parts of the world and continue to affect the daily lives of many thousands of people, there is a relative dearth of published information on the plight, health status and challenges facing such populations. This is largely due to the fact that in countries affected by armed conflict, local medical and health research efforts are often compromised by limited infrastructure, lack of human resources (both in terms of numbers and capacity) and insecurity. Medical and health policy research is thus limited. When it is done, it is often conducted by international non-governmental and humanitarian aid organizations who are the main actors on the scene.

There are several issues of ethical concern specific to the design and conduct of research in conflict settings.

First, many developing countries with fragile political climates, and particularly conflict-affected countries, often lack capacity to provide adequate scientific and technical guidance and monitor research ethics. As a consequence, international agencies may apply divergent ethical standards, some of which may not be in accordance with international human rights or humanitarian law [1]. (This has been a particular concern with respect to the activities of pharmaceutical companies that have been accused of deliberately circumventing their own national ethical standards and taking advantage of loosely enforced or poorly elaborated ethical guidelines in developing countries [2].)

Second, humanitarian organizations who might need to conduct qualitative and quantitative surveys as part of their relief operations are often not trained in the ethical appraisal of research as this is often not perceived as being part of their core mandate.

Third, lack of infrastructure and human resources, as well as the presence of violence, can limit both access to populations over time and the ability to conduct research. As a consequence, conventional research methodologies when applied to conflict settings without due adaptation may compromise the quality of the eventual results. For example, recent Iraq mortality surveys have been heavily criticized on the basis that sampling methods assumed homogenous distributions of violence and static makeup of households, which are uncharacteristic of conflict settings [3,4]. As a consequence, study results are similarly compromised in their ability to inform and impact policy.

Finally, international governmental and non-governmental organizations may face political pressure and barriers to research. In particular, the dissemination of sensitive findings might culminate in expulsion of organisations from conflict areas or penalisation of individuals or both. An example is given by the imprisonment in 2005 of a representative of a humanitarian organisation for the publication of data exposing the extent of sexual violence in Darfur [5]. Humanitarian organizations that have reported on human rights abuses and medical/nutritional emergencies in certain countries have been forced to withdraw from those countries or have been expelled.

Despite these issues, there is a clear justification and necessity to conduct research in conflict zones in order to improve knowledge of specific health interventions and their outcomes and bring to light the plight of populations caught in conflict. Not striving to do so may contribute to their vulnerability and add to complacency among those who are responsible or contribute to their unfortunate plight.

This paper discusses some of the main considerations for organisations and individuals engaged in research in conflict settings, and provides guidance on the main ethical principles to be applied.

## Why do research in conflict settings?

Humanitarian agencies such as Médecins Sans Frontières (MSF) are undertaking an increasing amount of research in conflict settings, often with the support of academic institutes and actors. For example, the number of peer-reviewed articles published by MSF has increased 10-fold in the last 7 years; this includes research in conflict-settings such as Chad, Liberia, Chechnya and the Democratic Republic of Congo ().

There are several reasons for specifically undertaking research in conflict settings. The main ones include: reporting on the health and humanitarian consequences of conflict; investigating the feasibility and effectiveness of specific interventions; and validating models of delivery.

### Reporting on the health and humanitarian consequences of conflict

Retrospective mortality surveys are the most common method applied in conflict settings to estimate the most important consequences of conflict on health status as indicated by standardized "death rates". The death rates in under five and over five age groups are often used as the most important parameter to judge the necessity of emergency humanitarian action [6]. Such techniques have generated valuable information regarding the impact of a number of conflicts, including those in DRC [7], Iraq [8], Myanmar [9], Sudan [10] and Congo Brazzaville [11]. Information on the nature of trauma or surgical wounds, levels of malnutrition, and surveillance of epidemiological data allow proper needs-assessments, the need for specific nutritional interventions or specific action to control specific epidemics.

Despite the availability of proposed methodologies [12] there is considerable variance in the quality of the implementation. Given the highly-politicized nature of conflict this can lead to debate about the legitimacy of the research findings [13]. This can result in a particular mode of action or inaction on the part of the warring parties, and have consequences in terms of the application of international humanitarian and human rights law.

Statistical measures alone will fail to capture the full impact of modern warfare on individual/population health and human rights, and non-consequentialist outcomes such as the gathering of personal witness testimony (from victims of injustice) or bearing witness (in the case of care providers) form an important part of the overall picture and provide evidence for action and redress [14]. Testimony also plays an important part in contributing to 'historical truth' and can contribute to post-conflict reconciliation efforts [15]. Other methodologies are emerging in response to the recognition that the impact of conflict on civilian populations should not be assessed by mortality rates alone. Mental health surveys are increasingly applied in conflict settings to estimate the consequences of violence and such survey results have been published for conflicts in a diversity of settings, including Sri Lanka [16], Sierra Leone [17], and Chechnya [18]. These surveys generally include questions on the experience of conflict, together with an assessment of general and psychosocial health. There are a number of specific challenges associated with conducting such research in conflict settings, in particular the validity of the survey tools used [19].

### Investigating specific interventions

Certain interventions are designed to support populations caught in conflict and research cannot therefore be easily conducted anywhere else. Examples include psychosocial interventions for psychosocial trauma-mitigation [20], micronutrients to manage anaemia in malnourished populations displaced by conflict [21], and trauma surgery [22]. In other instances, a study may be undertaken on an intervention for a condition that is not specifically conflict-related, but which predominates in or is exacerbated by conflict settings. The control of human African trypanosomiasis in the Republic of Congo is one such example [23]. For some contexts, general evidence isn't enough to inform policy change and local evidence is required: the introduction of artemisinin-based combination therapy for the treatment of malaria is an example where MSF and other agencies were involved in the conduct of a numerous drug efficacy studies, including in conflict-affected areas, as a precondition to being able change drug regimens [24].

### Validating models of delivery

Humanitarian agencies are concerned about improving approaches to the delivery of care. Research is particularly relevant in this area to assess the feasibility and provide a sound evidence-base for promoting new approaches. Examples include community-based therapeutic feeding for malnourished infants [25], the delivery of tuberculosis treatment in unstable settings [26], and HIV care and treatment for populations caught in conflict [27].

Epidemiological approaches have contributed to increasing accountability within the humanitarian sector, with most organisations today referring to agreed indicators outlined by such initiatives as the Sphere Project to guide their interventions [6]. Such minimum standards aim to improve the overall efficiency and effectiveness of the relief. Such indicators however cannot remain static and need to evolve dynamically over time as there might be variations in the epidemiology of disease, socio-demographic patterns and the environment where conflict occurs [28].

## Core elements of research in conflict settings

### Types of evidence

Traditionally, evidence-based medicine relies on the collection of quantitative data through epidemiological methodology. More broadly, evidence is the basis for inferences and the generation and testing of hypotheses and can take many forms. Humanitarian action relies on a broad range of evidence that includes quantitative data gathered from surveys and routine programme monitoring and qualitative information gathered via questionnaires, narrative accounts, policy analysis and expert inputs [14].

The type and quality of evidence gathered should be tempered by the circumstances being described and ability and limitations in undertaking specific research. Human rights violations are difficult to capture in numbers and may better be described through a qualitative approach through for example the collection of personal witness testimony. In terms of quantitative data collection, it should be recognized that the implementation of randomized-controlled trial designs in unstable settings is rarely feasible. In such circumstances rapid assessments may be the only feasible option for data collection. Nevertheless, provided clear methods are followed and measurements are carefully conducted, rapid approaches can still yield scientifically valid data. Moreover, it may be the only data available that can guide assessments of immediate needs and guide the implementation of relief efforts.

While there are inherent difficulties in collecting high quality data in conflict settings, there is an obligation to ensure that the research methodology being applied is of the highest standard, whether this is via a simple qualitative survey (gathering testimonies of refugees fleeing conflict) or a cohort study (most commonly the retrospective analysis of routinely collected data). The type of research methodology applied can influence both the scientific validity of the data collected and the requirements for ethical review.

### Scientific validity

The conditions of instability inherent to conflict settings create a number of barriers to undertaking high-quality scientific research. Basic data collection systems may be absent or poorly implemented; insecurity may limit movement and the ability to collect new data through surveys; the unpredictability of the setting may preclude study designs that require a large sample size or a long follow-up period; the displacement of populations will limit the potential for conducting prospective studies requiring return visits. Studies that require follow-up information on patients might also prove impossible.

Given such considerations, it is not surprising that most studies emanating from conflict settings are dependent on routinely collected data or rapid surveys. There is nevertheless still scope to improve the study design, the implementation of studies and their analysis and reporting in conflict settings.

### Study design

The development of study protocols can be greatly improved through collaboration with groups outside the conflict setting. Literature reviews, protocol writing, and the testing and validation of questionnaires, can all be done in a stable setting with expert support. Validated data collection and survey tools can be prepared in advance so that such studies can be rapidly implemented.

The validity of simple observational studies can also be greatly improved by relatively straightforward modifications such as randomization in the sampling strategy and blinding of data analysts.

### Study Implementation

One major challenge to research done by humanitarian agencies is that the study team is composed of people who are also involved in the delivery of aid. Research rightly takes second-place to the provision of life-saving assistance, but the result is that often the research is poorly conducted or abandoned altogether. While this is understandable, it raises ethical concerns (engaging research participants without completing the research) and is a waste of resources. As far as possible, the initiation of research should be preceded by a feasibility analysis and resource commitment to see it through to completion. The designation of dedicated study personnel, including people trained in research methods, should be encouraged to avoid the diversion of human resources to other activities (recognizing that expansion of teams is not always possible in insecure settings). As expatriate turnover in conflict situations is high, involving and training a core group of national collaborating researchers would facilitate continuity of the research and would constitute a resource pool for the future. The possibility of identifying a pool of responsive academics or resource persons to help support specific tasks (such as protocol review or statistical analysis) could also be considered.

### Analysis and reporting

The analysis and reporting of data requires expertise that is often not found in those involved in aid delivery. The reliability of the data analysis can often be substantially improved by involving epidemiologists and/or statisticians who are capable of undertaking more robust analyses. Editorial skills should also be sought to assist in writing papers for peer-reviewed publications. Far too much valuable information lies in drawers of aid agency offices because nobody has the time nor the expertise to write up and publish the findings.

## Ethical requirements

The requirement for ethical review depends on the research methodology and the specific population involved. This would vary depending on if the research involves routine monitoring and evaluation, hypothesis testing, or clinical research.

### Routine Monitoring and evaluation

Routine monitoring and evaluation provides valuable data that can inform future interventions. Examples include the reporting of the provision of antiretrovirals in conflict [27], and prison settings [28], improving approaches to the management of severe malnutrition [29] and outcomes of treatment of infectious diseases that predominate in conflict settings [30]. Humanitarian agencies are first and foremost care providers, and their contribution to the evidence base is most often done in this way because the implications in terms of additional resources are minimal, and analyses can be done post hoc, once the emergency has passed.

The generation of such information does not normally require ethical review [31]. But this does not mean that such work is free from ethical considerations. In particular, if data derived from routine assistance programmes to vulnerable groups is reported, particular care must still be taken to ensure their vulnerability is not further exploited, or worsened [32]. Confidentiality issues, anonymity of personal identity within datasets, and the right to refuse participation are all fundamental ethical considerations that cannot be compromised.

Another consideration when publishing routinely collected data is the possible harm to an individual's autonomy who had not been informed of, or given the possibility of consenting to the use of their clinical data. This has been debated in submissions for publication of such data relating to the provision HIV/AIDS care in prisons in resource-limited settings [31] and the treatment of human African trypanosomiasis in a conflict-affected country [33]. The view taken by the journal ethics committees in both instances was that the benefit of making the data available outweighed any potential harms of publishing such anonymized data.

What is arguably a far greater consideration is the obligation to report such data, particularly where this relates to a new approach to delivering care as the experience might have much wider implications for policy and practice. Humanitarian agencies will often use routinely collected data to inform their own programmes but will not invest in the resources and expertise to analyse such data further and share this information in the public domain, often because the emergency has passed and the focus has moved elsewhere [34]. In its most extreme form, the withholding of information or its sub-optimal utilisation when it could broadly serve to improve individual and public health outcomes can be viewed as a violation of the common good. This notion is based on the argument that solidarity and the recognition that health, human rights, economic opportunities, and development are all intimately linked [35]. In this way, information gathered for specific programme purposes may in fact have the potential to influence a much broader set of concerns, and this potential must be considered and exploited wherever possible.

### Hypothesis testing

If a research hypothesis is to be tested – even by interrogating routinely collected data – new knowledge is being generated and there might be ethical considerations. Deciding on whether ethical review or oversight is needed or not is a particularly grey area to assess for humanitarian actors who are involved in the routine delivery of care and not familiar with the research process. In general, it is recommended that all studies destined for public dissemination be subjected to ethical review and advice until routine standards are set.

### Clinical studies

Clinical studies clearly require ethical review, but such studies ought to be exceptional. Agencies considering undertaking clinical studies in conflict settings should be challenged to justify why the study cannot be undertaken in non-conflict settings. Examples of such justifiable exceptions include the study of new medicines to treat human African trypanosomiasis, a parasitic disease whose spread is directly related to conflict and the disruption of vector control programmes [36].

## Ethical challenges to research in conflict settings

### General considerations

Ethical norms and standards for medical research have been established since Nuremberg and are elaborated in such documents as the CIOMS guidelines [37]. (see Additional File 1) A number of issues have emerged as being of particular relevance to developing countries but are also applicable to conflict settings [38]. These are outlined below.

#### Informed consent

Informed consent is an integral part of acknowledging an individuals' autonomy and protecting those with diminished autonomy (eg prisoners, refugees, children). In research this translates into taking practical steps to respect confidentiality and ensure privacy. The process of obtaining informed consent must be sensitive to the norms, customs and sensitivities of the local environment. A high degree of illiteracy, or mistrust of authority may mean that signing a consent form is meaningless or even dangerous. At worst, the need for informed consent has been overlooked entirely [2]. In humanitarian crises, researchers are also often the providers of assistance, and particular care must be taken to ensure that consent or refusal to participate is in no way interpreted as being linked to the provision of assistance [38].

#### Research design

Particular problems have arisen with respect to the application of standards of care: should this be set according to the country in which the research is being undertaken, or according to the country of origin of the researchers, or the best available standard-of-care worldwide? According to the Declaration of Helskinki [39]: "The benefits, risks, burdens and effectiveness of the new method should be tested against those of the best current prophylactic, diagnostic and therapeutic methods." However, the definition of 'best current' has been debated, particularly in countries in which the international gold standard will be unattainable for many years to come [40].

#### Ethics review

The capacity of ethics review boards in developing countries is highly variable; some countries in conflict have no ethics review board at all. To what extent researchers must seek approval from other ethics boards, and to what extent this is practical or appropriate, is not always clear. In any case when there is a need for ethical scrutiny and no local ethical board is available, ethical review by a board outside the setting is still better than nothing.

#### Benefits to participants

Maximizing the benefits of research for participants requires that researchers provide practical guarantees that the study population will benefit in some way from the research being conducted. The Declaration of Helsinki states that: "At the conclusion of the study, every patient entered into the study should be assured of access to the best proven prophylactic, diagnostic and therapeutic methods identified by the study." This is not always possible. For example, if the treatment for a chronic disease is being tested in a country where no such treatment is provided, should the researchers be obliged to provide such treatment to study participants and if so, for how long? This was a very real challenge to those implementing antiretroviral therapy in resource-poor settings once this started to became more widely available as from 2001.

### Specific Considerations

In addition to the above general concerns, there are a number of concerns that are of particular importance in conflict settings.

#### Vulnerability

Populations exposed to conflict have heightened vulnerability resulting from physical and mental distress, the collapse of normal coping mechanisms, and deliberate targeting (for example because they belong to a particular ethnic group). They may be subject to multiple human rights abuses [1]. The potential for exploiting a situation of "differential power" which could lead to denying or compromising the rights of individuals is difficult to control.

Asking someone to talk about experiences that were frightening, humiliating or degrading can increase the level of trauma associated with the event. Efforts should be made to assess individuals in a particular group who are particularly vulnerable and as far as possible excluded them from research. Examples of such individuals may include victims of recent violence, groups at high risk of stigmatization and minors. In particular, gathering information from victims of sexual violence requires a heightened level of sensitivity to a range of issues such as religious beliefs, cultural and social values, the legal environment, and gender issues [41]. In practice, this could mean seeking information from secondary sources. Extensive interviewing with victims of violence should be carried out only in very exceptional circumstances, and by trained investigators with a clear methodology and possibilities for referral for psychosocial support.

There may be a need for an increased level of confidentiality of study data in situations where even the simplest information (household composition, age of males) could provide information to support deliberate targeting of individuals/groups by perpetrators of violence.

Finally, being mindful of the population's physical vulnerability means taking into consideration the timing and duration of assessment to avoid disruption of essential service delivery. This means, for example, avoiding the conduct of interviews during the time when food distributions occur (a problem that was recently observed to occur in Darfur).

#### Necessity of conducting research in conflict settings

It can be questioned to what extent populations placed under such stress should be subjected to research – it has for example been argued that refugees for example should never be subjected to medical research [42]. However, this argument rests on the assumption that optimal knowledge exists, and further improvements in delivery are unnecessary, an assumption that is difficult to accept. Civilians caught in conflict have basic rights to medical and other assistance, and those involved in the delivery of such assistance have a duty to optimize the efficiency and relevance of their work. Indeed, it may be unethical not to generate knowledge intended to evaluate or improve delivery of services in such contexts. For example, the provision of antiretroviral therapy to people in conflict settings was recommended against [6] until pilot programmes proved that it could be done effectively (Culbert et al 2007). Categorically excluding certain vulnerable groups from research altogether may contribute to their vulnerability by preventing the design and improvement of interventions specifically tailored to their needs, and may in fact violate principles of social justice by leaving them worse off than they otherwise would have been.

Research in conflict settings is necessary to answer important questions specifically related to health care of populations caught in conflict. But it must also be acknowledged that researchers may have a personal interest in conducting research in a conflict setting when in fact the research question could be posed in any number of more stable settings [43]. The necessity of conducting research in conflict settings to answer questions that could just as easily be answered in a non-conflict setting should be questioned, both because of the heightened vulnerability of populations caught in conflict, and the particular logistical and security (and so cost) implications of undertaking research in conflict settings.

Finally, the multitude of international organizations that are often present in disaster settings and gathering information for various reasons – operational planning and evaluation, advocacy, and operational research – can lead to an over-assessment of populations: for example in Darfur it has been reported that some communities have been surveyed more than five times [44].

#### Feasibility of implementing research and delivering its benefits

Conflict settings are by definition dynamic and subject to rapid deterioration. This can have serious implications for the implementation of research. Essential supplies may be limited; structures in which research is being conducted (eg hospitals and health centres) may be destroyed; data collection systems may be disrupted; research teams may have to evacuate and the study populations may become displaced. The design and conduct of research must take into account the dynamic environment in which the research will be conducted to maximize the chances that such research will be completed.

The feasibility of delivering benefits of research should also be carefully considered. The 2002 CIOMS Guidelines state that "the research is responsive to the health needs and the priorities of the population or community in which it is to be carried out; and any intervention or product developed, or knowledge generated, will be made reasonably available for the benefit of that population or community" [37]. However, this may be difficult to guarantee in a conflict setting where both investigators and participants may be displaced due to insecurity.

## An ethical framework for research in conflict settings

In June 2003 the international aid agency MSF established an independent ethics review board for the assessment of research conducted in settings where it works, many of which are conflict or post-conflict settings. This independent ethics review board developed a set of practical benchmarks for the ethical conduct of research, based on an ethical framework drafted by some members of the US National Institutes of Health [38] (Table 1). The framework was tested over a period of 18 months to assess its utility and feasibility. In the last 5 years the framework has since been used to review over 50 proposals, and while a review of the specific relevance of each benchmark is warranted, the framework has been found to be overall well adapted to the settings where MSF conducts research.

**Table 1:** Ethical framework for research: benchmarks used by MSF

Benchmark	Practical Interpretation	Considerations
**Collaborative Partnership**	Researchers should engage in partnership with national and/or international research institutions as relevant and appropriate. Such collaboration should contribute to developing the capacity for researchers and health policymakers to become full and equal partners in the research enterprise.	May not be possible due to the absence of partners or the partisan nature of certain groups that prevent a neutral engagement. However, there should be a demonstrated effort to seek reliable partners.

**Community engagement**	Researchers should respect the community's values, culture, traditions, and social practices; involve the community in the design and implementation of research through a consultative process; and share fairly any financial and other rewards of the research.	Traditional community organisation may be disrupted by conflict. Values, cultures, traditions and practices may all be disturbed by conflict. There may be risks of bias in politically polarized environments.

**Social value**	Beneficiaries should be clearly specified, and importance of the health problems being investigated and the prospect of value of the research for the beneficiaries made clear. Mechanisms to enhance the social value of the research should be established to ensure that knowledge is disseminated and ensuring the community benefits from the knowledge generated. Efforts should be made to avoid diverting resources from health services for the conduct of research.	Transient nature of conflict prevents guarantees that research participants will benefit directly.

**Scientific Validity**	Research design should optimize possibilities of achieving the social value requirements. Research should be feasible given the social, political, and cultural environment and with sustainable improvements in the local health care and physical infrastructure. Finally, it should be of sufficient quality (eg of sufficient sample size) to yield reliable information.	Volatile nature of conflict can disrupt conduct research (eg sampling constrained by security; evacuations prevent achieving initial sample size)

**Fair selection of participants**	Study population should be selected in such a way as to ensure scientific validity of the research and minimize the risks of the research. This means formulating clear inclusion and exclusion criteria and identifying and protecting vulnerable groups.	Initial selection/recruitment may need to be adjusted due to population displacement

**Favourable Harm-Benefit Ratio**	Protocol should clearly assess potential harms and benefits to the study participants and the harm-benefit ratio for the community.	Given the complex cultural and political context of conflict settings, community members may need to be involved in such assessments.

**Informed consent**	Study community should be involved in establishing appropriate recruitment procedures and incentives for the participants. Consent procedures should be acceptable and practical within the study community. All information should be disclosed in culturally and linguistically appropriate formats. Participants must fully comprehend the research objectives and procedures. This may mean allowing adequate time for discussion about the information received with members of the community or family before deciding on consent. It should be made clear that participants are free to refuse or withdraw from the research at any stage without penalty.	The provision of incentives should be carefully considered to gain consent from vulnerable populations Particularly important when the organisation undertaking the research is also engaged in the delivery of humanitarian assistance.

**Respect for Recruited Participants and Study Communities**	Procedures should be established to protect the confidentiality of recruited and enrolled participants (including biological samples). Enrolled participants should be provided with relevant new information that arises in the course of the research. Medical conditions, including research related injuries, of enrolled participants should be monitored and care provided that is at least as good as existing local norms. Participants and the study community should be informed of the results of the research. Harms and wrongs should be assessed to minimize the risk of exploiting research participants.	May be divergence between 'local' and 'acceptable' norms of care. Conflict-related displacement of research organisation or population may prevent feedback.

**Independent review**	Public accountability of the research should be ensured through scientific and ethical review according to international standards. This may require the engagement of international as well as local ethics review boards.	Complicated in oppressive settings if publication research findings may cause problems to researchers or the organisation

The main principles elaborated in this framework are: (i) collaborative partnership (ii) community engagement (iii) social value (iv) scientific validity (v) fair selection of study participants (vi) favourable harm-benefit ratio (vii) informed consent (viii) respect for recruited participants and (ix) independent review. The interpretation of these principles, and considerations for their implementation in conflict-settings, are summarized in Table 1.

## Conclusion

Despite arguments that it is unethical to conduct research on vulnerable populations caught in conflict, health research is essential to achieving the goals of promoting and enhancing the delivery of life-saving interventions.

Conflict settings are characterized by instability and rapidly change in circumstances and pose major challenges to the conduct of research. Given these challenges and concerns, those engaged in research should first reflect on the necessity of conducting research in such settings, and the feasibility of seeing the research through to completion.

There is much scope to improve the quality of research methodologies implemented in conflict settings through validation of survey tools, the establishment of clear protocols, and training in a broader use or research methodologies that can be applied in such settings. This implies training for humanitarian actors engaged in research methods, and greater collaboration with experts to support the design and analysis, and potentially also the implementation of research. Adequate resources should be made available to support research in conflict settings, including the potential to engage specialized staff from academic or other settings to support research, either directly or indirectly. Finally, concepts of scientific validity may need to be modified for conflict situations.

There are a number of particular ethical issues associated with the conduct of research in conflict settings; these relate primarily to the vulnerability of participants. Efforts are needed to improve the knowledge and capacity of international organizations about international ethical guidelines associated with medical and health policy research. At the same time, ethics review boards need to engage people with established experience in the specific demands of conducting research in conflict settings, and those ethical considerations that require special attention. Research approaches should be informed by and consistent with international human rights and humanitarian laws as well as ethical guidance.

Finally, greater attention should be paid to the dissemination of the results of research, to ensure that essential information is shared as widely as possible to the benefit of all those engaged in improving the wellbeing of populations caught in conflict.

## Competing interests

The authors declare that they have no competing interests.

## Authors' contributions

All authors contributed to the writing, revision and supportive literature reviews in the preparation of this manuscript, and read and approved the final manuscript.

## Supplementary Material

Additional file 1 **Further reading**. Additional resources related to research ethics in conflict settings. Click here for file
