# Supplementary material for: Ethics of conducting research in conflict settings
Source: Confl Health. 2009 Jul 10;3:7. doi: 10.1186/1752-1505-3-7 (PMC2717053; doi:10.1186/1752-1505-3-7)
Supplement: Additional file 1 — Further reading. Additional resources related to research ethics in conflict settings. [file 1752-1505-3-7-S1.doc]

**Additional file 1: Further reading**

**Description: Additional resources related to research ethics in conflict settings**

The Belmont Report: Ethical Principles and Guidelines for the Protection of Human Subjects of Research. The (US) National Commission for the Protection of Human Subjects of Biomedical and Behavioral Research, Office for Protection from Research Risks, DHSS, National Institutes of Health 1979

Available at: http://ohrp.osophs.dhhs.gov/humansubjects/guidance/belmont.htm

Declaration of Helsinki: Recommendations guiding physicians in biomedical research involving human subjects

World Medical Association 1964 (with amendments 1975, 1983, 1989, 1996)

Available at: http://www.wma.net/e/policy/17-c_e.html

The Nuremberg Code. Trials of War Criminals before the Nuremberg Military Tribunals under Control Council Law No. 10. Nuremberg, October 1946–April 1949. Washington, D.C.: U.S. G.P.O, 1949–1953

Available at: http://www.ushmm.org/research/doctors/Nuremberg_Code.htm

International Ethical Guidelines for Biomedical Research Involving Human Subjects

WHO/ Council for International Organizations of Medical Sciences (CIOMS) 1993

Not available online but ordering information found at

Available at: http://www.who.int/dsa/cat98/ethic8.htm

International Ethical Guidelines for Ethical Review of Epidemiological Studies

WHO/ CIOMS 1991. Available at: http://www.cdc.gov/od/ads/intlgui3.htm

Operational guidelines for ethics committees that review biomedical research

The WHO-UNDP-World Bank Special Programme for Research and Training in Tropical Diseases (TDR); available online in English, French, Spanish, German, Turkish, Thai & Russian. Available at: http://www.who.int/tdr/publications/publications/ethics.htm
